# Identification of a Tumor Microenvironment-relevant Gene set-based Prognostic Signature and Related Therapy Targets in Gastric Cancer

**DOI:** 10.7150/thno.47938

**Published:** 2020-07-09

**Authors:** Wang-Yu Cai, Zi-Nan Dong, Xiao-Teng Fu, Ling-Yun Lin, Lin Wang, Guo-Dong Ye, Qi-Cong Luo, Yu-Chao Chen

**Affiliations:** 1 Department of Gastrointestinal Surgery, Zhongshan Hospital, Xiamen University, Xiamen, Fujian, China.; 2 Institute of Gastrointestinal Oncology, School of Medicine, Xiamen University, Xiamen, Fujian, China.; 3 Pancreatitis Center, The First Affiliated Hospital of Wenzhou Medical University, Wenzhou, Zhejiang, China.; 4 State Key Laboratory Breeding Base of Marine Genetic Resources; Key Laboratory of Marine Genetic Resources, Third Institute of Oceanography, Ministry of Natural Resources, Xiamen, Fujian, China.; 5 Laboratory of Xiamen Cancer Center, The First Affiliated Hospital of Xiamen University, Xiamen, Fujian, China.; 6 Department of Pathology, School of Medicine, Jinan University, Guangzhou, Guangdong, China.

**Keywords:** gastric cancer, prognostic signature, machine learning, tumor microenvironment, targeted therapy

## Abstract

**Rationale:** The prognosis of gastric cancer (GC) patients is poor, and there is limited therapeutic efficacy due to genetic heterogeneity and difficulty in early-stage screening. Here, we developed and validated an individualized gene set-based prognostic signature for gastric cancer (GPSGC) and further explored survival-related regulatory mechanisms as well as therapeutic targets in GC.

**Methods:** By implementing machine learning, a prognostic model was established based on gastric cancer gene expression datasets from 1699 patients from five independent cohorts with reported full clinical annotations. Analysis of the tumor microenvironment, including stromal and immune subcomponents, cell types, panimmune gene sets, and immunomodulatory genes, was carried out in 834 GC patients from three independent cohorts to explore regulatory survival mechanisms and therapeutic targets related to the GPSGC. To prove the stability and reliability of the GPSGC model and therapeutic targets, multiplex fluorescent immunohistochemistry was conducted with tissue microarrays representing 186 GC patients. Based on multivariate Cox analysis, a nomogram that integrated the GPSGC and other clinical risk factors was constructed with two training cohorts and was verified by two validation cohorts.

**Results:** Through machine learning, we obtained an optimal risk assessment model, the GPSGC, which showed higher accuracy in predicting survival than individual prognostic factors. The impact of the GPSGC score on poor survival of GC patients was probably correlated with the remodeling of stromal components in the tumor microenvironment. Specifically, TGFβ and angiogenesis-related gene sets were significantly associated with the GPSGC risk score and poor outcome. Immunomodulatory gene analysis combined with experimental verification further revealed that TGFβ1 and VEGFB may be developed as potential therapeutic targets of GC patients with poor prognosis according to the GPSGC. Furthermore, we developed a nomogram based on the GPSGC and other clinical variables to predict the 3-year and 5-year overall survival for GC patients, which showed improved prognostic accuracy than clinical characteristics only.

**Conclusion:** As a tumor microenvironment-relevant gene set-based prognostic signature, the GPSGC model provides an effective approach to evaluate GC patient survival outcomes and may prolong overall survival by enabling the selection of individualized targeted therapy.

## Introduction

Gastric cancer (GC) remains one of the most prevalent malignant diseases worldwide and was the third leading cause of cancer mortality in 2019 1, 2. The incidence rate of gastric cancer highly varies among regions, with more than 70% of cases occurring in developing countries and more than 50% of cases occurring in Eastern Asia 3. Although the prevention and treatment of *Helicobacter pylori* and Epstein-Barr virus (EBV) infection have decreased its incidence and mortality rates, the 5-year survival rate of GC patients is still lower than 30% 3, 4. Due to the genetic heterogeneity and difficulty in early-stage screening, the prognosis of GC patients is adversely affected by the limited therapeutic effects in both locally advanced and metastatic settings 5, 6. Chemotherapy and targeted drugs do not achieve precise treatment, often leading to poor outcomes 4. The detection and analysis of tumor prognostic markers are of great significance to evaluate tumor progression, predict therapeutic efficacy, reduce the recurrence rate and mortality, and prolong survival.

Strategies to identify the subset of GC patients likely to have poor survival and high mortality are needed for additional clinical therapy. TNM staging has been widely used for determining GC prognosis 7-9 but is limited by the variations among patients with the same tumor stage. Studies have shown that the treatment response and survival rate of GC patients depend not only on tumor staging but also on heterogeneous and epigenetic molecular features 10-12. Biomarkers, especially gene expression in tumor tissues, are reliably related to cancer prognosis and survival 13-16. Nevertheless, further analysis and validation in larger, independent cohorts in combination with more potential markers are essential prior to application in a clinical setting. The availability of large-scale public cohorts with gene expression data and well-developed biological databases provide opportunities to identify a more generalized prognostic signature for gastric cancer. Recently, machine learning, as a branch of artificial intelligence (AI), has been employed to establish prognostic classification models for outcome and therapy prediction in individual cancer patients 17-20. For example, via machine learning, Tang *et al.* demonstrated that gene expression data can be used for valid predictions of nasopharyngeal carcinoma distant metastasis and survival 21. Therefore, applying machine learning and statistical techniques to GC prognostication and outcome prediction based on large and comprehensive datasets may provide a novel strategy for applying personalized medicine in gastric cancer.

The tumor microenvironment (TME), consisting of extracellular matrix (ECM), stromal cells, immune/inflammatory cells, and secreted factors, has been revealed to be highly correlated with cancer progression and therapeutic responses 22-24. Evaluation of all TME components based on machine learning has been utilized to predict survival in gastric cancer and develop an effective therapeutic strategy. By estimating TME cell infiltration patterns in gastric cancer patients, Zeng *et al*. defined three TME phenotypes and developed a quantifiable TMEscore as a prognostic biomarker to predict immunotherapeutic benefits 25. In another study, Kim *et al*. revealed that survival outcomes were better in GC patients with a higher density of CD3(+) cells within the TME than in those with a lower density of CD3(+) cells 26. Therefore, TME-related gene set-based prognostic signatures have potential to be applied in gastric cancer.

In this study, we integrated multiple cohorts with gene expression data to develop and validate an individualized gene set-based prognostic signature for gastric cancer (GPSGC). Furthermore, the relationship between the GPSGC risk score and the tumor microenvironment was analyzed to explore survival mechanisms and therapeutic targets related to the GPSGC score. Finally, we used gastric cancer tissue microarrays for experimental verification to prove the stability and reliability of the GPSGC model and therapeutic targets.

## Methods

### GC gene expression data

We systematically searched for gastric cancer gene expression datasets that were publicly available and reported full clinical annotations. As the mortality of patients with an overall survival (OS) of <30 days may be due to other factors, these patients and those without survival information were excluded from further evaluation. For this study, we gathered five cohorts with a total of 1699 patients with gastric cancer (Table S1): TCGA-STAD, ACRG/GSE62254, GSE15459, GSE26253 and GSE84437.

Gene expression data (FPKM normalized) and the corresponding clinical datasheets for GC tissue samples and adjacent normal tissue samples of 342 patients in The Cancer Genome Atlas (TCGA) were downloaded from Genome Data Commons (https://portal.gdc.cancer.gov) (up to April 1, 2019). For the TCGA dataset, RNA-sequencing data (FPKM values) were transformed into transcripts per kilobase million (TPM) values, and then z-score normalization was performed.

The raw data from the microarray datasets generated by Affymetrix and Illumina platforms were downloaded from Gene Expression Omnibus (GEO; https://www.ncbi.nlm.nih.gov/geo/) (up to April 1, 2019). We obtained four additional datasets, GSE62254/ACRG, GSE15459, GSE26253, and GSE84437, which contained more than 80 gastric cancer patients with survival information in each cohort. The raw data for the dataset derived from an Affymetrix platform were processed using the RMA algorithm for background adjustment in the Affy software package. RMA was used to perform background adjustment, quantile normalization, and final summarization of oligonucleotides per transcript using the median polish algorithm. The raw data from Illumina were processed using the lumi software package. Finally, z-score normalization was performed for all the gene expression data.

### Prognostic model establishment

The gene expression differences between gastric cancer tissues and adjacent normal tissues were compared using the limma package in R software 3.3.1, and genes with |log fold change| > 1 and Benjamini-Hochberg-adjusted *P* < 0.01 were considered to be significant differentially expressed genes (DEGs). Survival analysis associated with these DEGs was performed by Kaplan-Meier analysis, and univariate Cox regression analysis using the 'survidiff' function in the survival package of R 3.3.1 was performed with *P* < 0.05 as the significance threshold (Table S2). Subsequently, stepwise Cox regression was used to calculate the results of the model by adding one gene at a time, so as to determine whether the newly added gene could significantly improve the accuracy of the results. Stepwise Cox regression can not only remove the collinear genes but also find the best combination of genes and establish the multivariate Cox regression model. Finally, an optimal risk assessment model was constructed utilizing the regression coefficients derived from stepwise Cox regression multivariate analysis to multiply the expression level of each marker gene. X-tile 3.6.1 software (Yale University, New Haven, CT, USA) was employed to determine the best cutoff for GC patients classified as low risk and high risk. The log-rank test and Kaplan-Meier survival analysis were used to assess the predictive ability of the prognostic model.

### TME characterization analysis

For TME subcomponent analysis, ESTIMATE, a method that uses gene expression signatures to infer the fraction of stromal and immune cells in GC samples, was utilized to determine stromal and immune scores via ssGSEA (Table S3).

For TME cell type analysis, xCell, a method that uses gene expression signatures to infer the proportions of 64 immune and stromal cell types in samples, was utilized to determine the enrichment score of each cell type via ssGSEA (Table S4).

For panimmune gene set analysis, gene set variation analysis (GSVA) was used to estimate the enrichment scores of 110 immunoregulation-related pathways in GC samples (Table S5).

For immunomodulatory gene analysis, the list of 78 immunomodulator genes summarized by experts in immune oncology in the TCGA immune response working group was utilized, and the expression levels of 60 detectable immunomodulatory genes were obtained in this study (Table S6).

### Multiplex fluorescent immunohistochemistry (mfIHC)

Tissue microarrays from 186 GC patients (HStmA180Su11, HStmA180Su13) were purchased from Shanghai Outdo Biotech Co., Ltd. (Shanghai, China). The studies were conducted in accordance with the International Ethical Guidelines for Biomedical Research Involving Human Subjects (CIOMS), and the research protocols were approved by the Clinical Research Ethics Committee of Zhongshan Hospital of Xiamen University.

For mfIHC staining, the Opal 7-color manual IHC kit (PerkinElmer, USA) was used according to the manufacturer's instructions. First, the concentration and order of the five antibodies were optimized, and the antibody panel was built based on single antibody-stained slides (VCAN antibody, 1:5000 dilution, Novus Biologicals, cat. no. NBP1-85432; CLIP4 antibody, 1:200 dilution, Abcam, cat. no. ab243532; MATN3 antibody, 1:200 dilution, Abcam, cat. no. ab238893; TGFβ1 antibody, 1:250 dilution, Abcam, cat. no. ab27969; and VEGFB antibody, 1:500 dilution, Absin, cat. no. abs136375). The slides were first baked at 63°C for 1 hour. Deparaffinization with xylene for 10 minutes in triplicate was followed by rehydration in 100%, 90%, and then 70% ethanol for 10 minutes each. Antigen retrieval was performed with a microwave. After incubation with 3% H_2_O_2_ (freshly made) for 10 min, the tissues were blocked in blocking buffer for another 10 min at room temperature. After antigen retrieval, slides were stained with antigen-specific primary antibodies followed by Opal Polymer (secondary antibody). Application of the Opal TSA fluorophore created a covalent bond between the fluorophore and the tissue at the site of the HRP. Each antigen retrieval step was performed using AR6 antigen retrieval buffer, which allowed for the removal of prior primary and secondary antibodies while the fluorophore remained covalently bonded to the tissue antigen. This allowed for the use of the same host species antibody while also amplifying the signal.

Imaging was completed using the Vectra Polaris Automated Quantitative Pathology Imaging System (PerkinElmer, USA). One image per core was captured at ×20 magnification. All cube filters were used for each image capture (DAPI, Opal 520, Opal 570, Opal 620, Opal 650, and Opal 690). The incorporated saturation protection feature was set at an exposure time of 250 ms.

The stained sections were scored by three pathologists who were blinded to the clinical characteristics of the patients. The scoring system was based on the intensity and extent of the staining. The staining intensity was classified as 0 (negative), 1 (weak), 2 (moderate) or 3 (strong). The staining extent was dependent on the percentage of positive cells (examined in 200 cells): 0 (< 5%), 1 (5%-25%), 2 (26%-50%), 3 (51%-75%) or 4 (>75%). According to the staining intensity and extent scores, the fluorescent immunohistochemistry results were classified as 0-1, negative (-); >1-4, weakly positive (+); >4-8, moderately positive (++) and >8-12, strongly positive (+++). To calculate the GPSGC risk score, the protein expression scores of VCAN, CLIP4 and MATN3 were normalized with a z-score (Table S7).

### Nomogram construction and evaluation

To validate whether the predictions of the prognostic model were independent of traditional clinical features for patients with GC, multivariate Cox regression analyses were conducted. We further used the coefficients of the multivariable Cox regression model and formulated nomograms using the rms package in R. Calibration curves were assessed graphically by plotting the observed rates against the nomogram predicted probabilities and a concordance index (C-index) was calculated to determine the discrimination of the nomogram via a bootstrap method with 1000 resamples.

### Statistical analysis

Statistical analyses were performed with R (version 3.3.1) and GraphPad Prism 8.0. Pearson correlation analysis was performed to determine the correlation between two variables. Survival analysis was performed using a log-rank test. *P* < 0.05 was considered statistically significant.

## Results

### Development and identification of the GPSGC in TCGA and ACRG training cohorts

**Figure 1:**
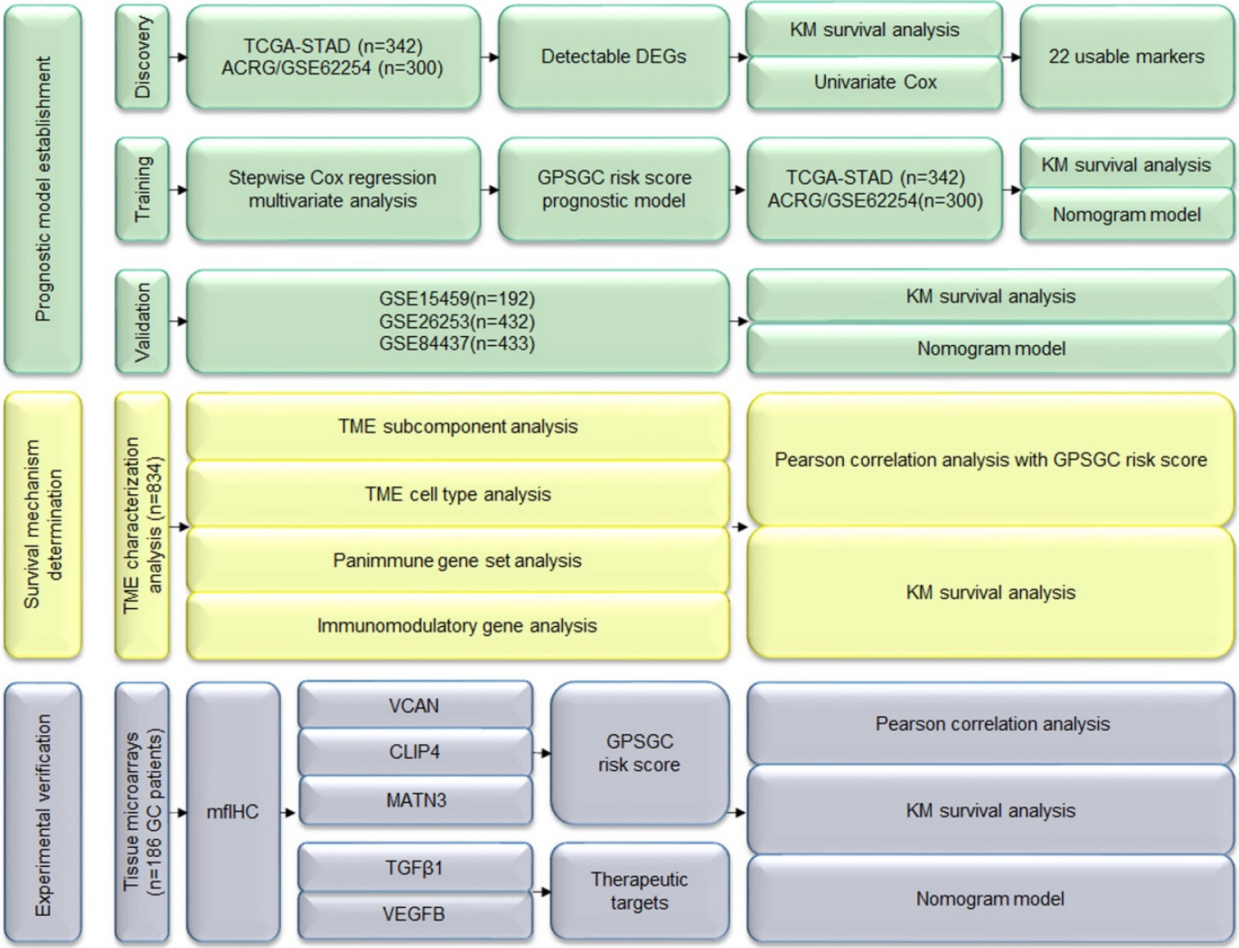
Study flowchart. DEGs: differentially expressed genes; GPSGC: gene expression-based prognostic signature for gastric cancer; KM: Kaplan-Meier; mfIHC: multiplex fluorescent immunohistochemistry; TME: tumor microenvironment.

In this study, we performed three major steps to establish accurate and reliable prognosis and treatment guidance for gastric cancer (GC): prognostic model establishment, survival mechanism determination and experimental verification. A total of 1699 patients with gastric cancer from 5 independent gene expression datasets (Table S1) and 186 GC patients with data from tissue microarrays and available clinical information were used for the analysis (Figure 1).

To develop the gene expression-based prognostic signature for gastric cancer (GPSGC), the gene expression differences between gastric cancer tissues and adjacent normal tissues in the TCGA-STAD training dataset were compared using the limma package, as removal of genes not detected in the ACRG training dataset, detectable differentially expressed genes (DEGs) were identified with the cutoffs |log fold change| > 1 and Benjamini-Hochberg-adjusted P < 0.01. Survival analysis via the Kaplan-Meier method and univariate Cox regression revealed that the expression levels of 22 DEGs were significantly associated with prognosis in the TCGA-STAD and ACRG cohorts, (P < 0.05; Table S2). Stepwise Cox regression multivariate analysis was further used to screen the best combination of genes and then construct an optimal multivariate Cox regression model, the GPSGC (Figure 1). A risk score was calculated for each patient using a formula derived from the expression levels of three genes weighted by their regression coefficient: risk score = (0.14121 * expression of VCAN) + (0.19095 * expression of CLIP4) + (0.13633 * expression of MATN3).

**Figure 2:**
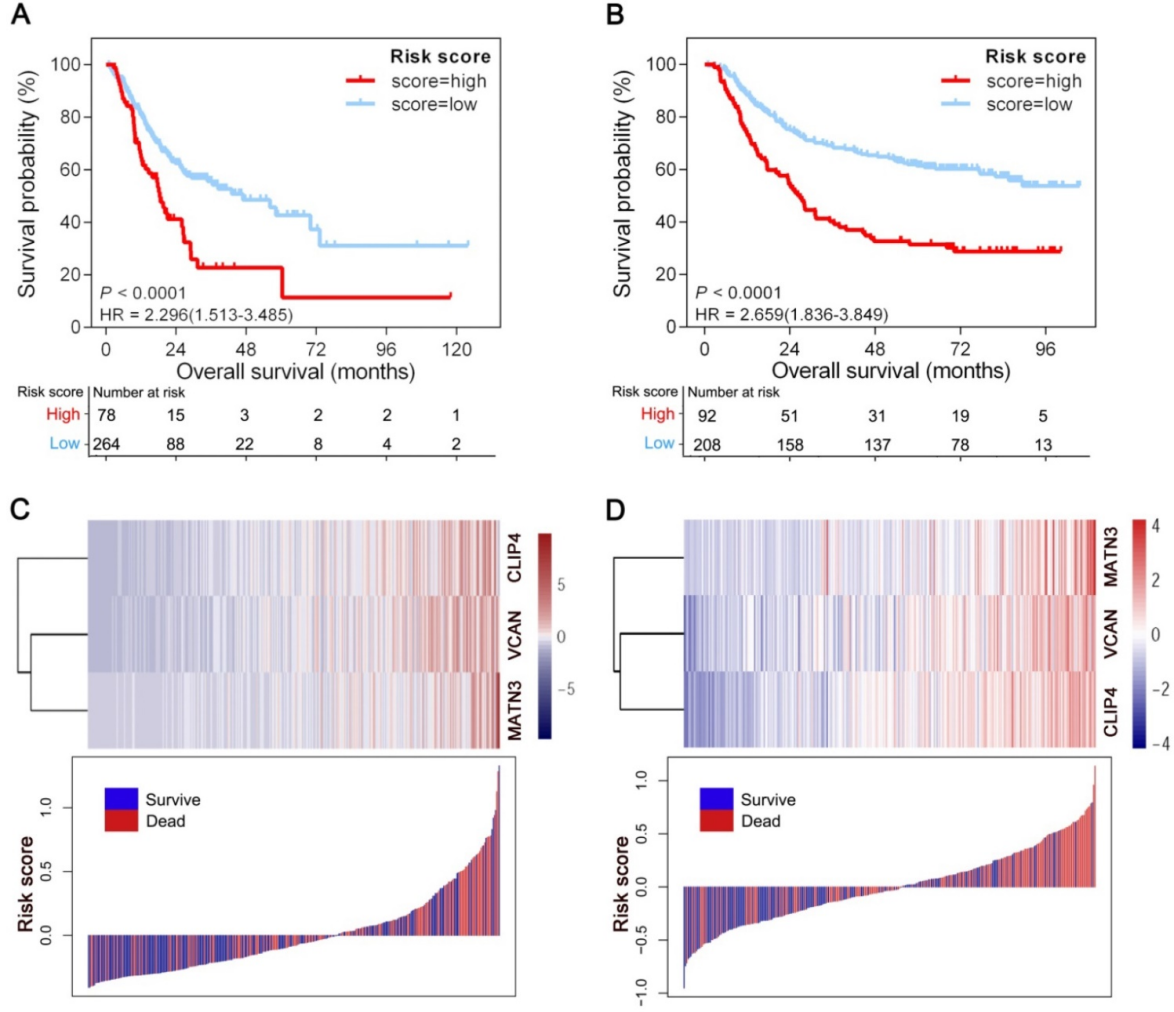
Generation of the GPSGC model from TCGA and ACRG training cohorts. (**A**) Kaplan-Meier curves for the high (n = 78) and low (n = 264) GPSGC risk score patient groups in the TCGA-STAD cohort. Log-rank test, *P* < 0.0001. (**B**) Kaplan-Meier curves for the high (n = 92) and low (n = 208) GPSGC risk score patient groups in the ACRG cohort. Log-rank test, *P* < 0.0001. (C-D) The relationships between the expression of three prognostic genes (upper) and GPSGC risk score distribution with survival status (bottom) in the TCGA-STAD (**C**) and ACRG (**D**) cohorts are shown; the X axis is sorted by GPSGC risk scores. Patients were divided into high-risk and low-risk groups with GPSGC risk score = 0.15 utilized as the cutoff value.

The optimal cutoff point (0.15) obtained from X-tile 3.6.1 software served as the cutoff to assign patients in the TCGA-STAD and ACRG cohorts into high- and low-risk groups. As shown in Figure 2A, patients with high risk scores (22.8%) had shorter overall survival (OS) (HR = 2.296; 95% CI: 1.513-3.485; *P* < 0.0001) than patients with low risk scores (77.2%) in 342 GC patients from the TCGA-STAD cohort. Consistently, the high-risk patients (30.7%) had a shorter OS than their low-risk counterparts (69.3%) (HR = 2.659; 95% CI: 1.836-3.849; *P* < 0.0001) in the ACRG cohort including 300 GC patients (Figure 2B). Also in line with these findings, the GPSGC score was more accurate for predicting short-term and long-term survival than individual prognostic factors (VCAN, CLIP4 or MATN3 expression level) in both cohorts (Figures 2A-B, S1A-F). The relationships between the expression of the three prognostic genes and GPSGC risk score distribution with survival status in the TCGA-STAD and ACRG cohorts are shown in Figure 2C and Figure 2D, respectively.

### Validation of the GPSGC in multiple GEO gastric cancer cohorts

**Figure 3:**
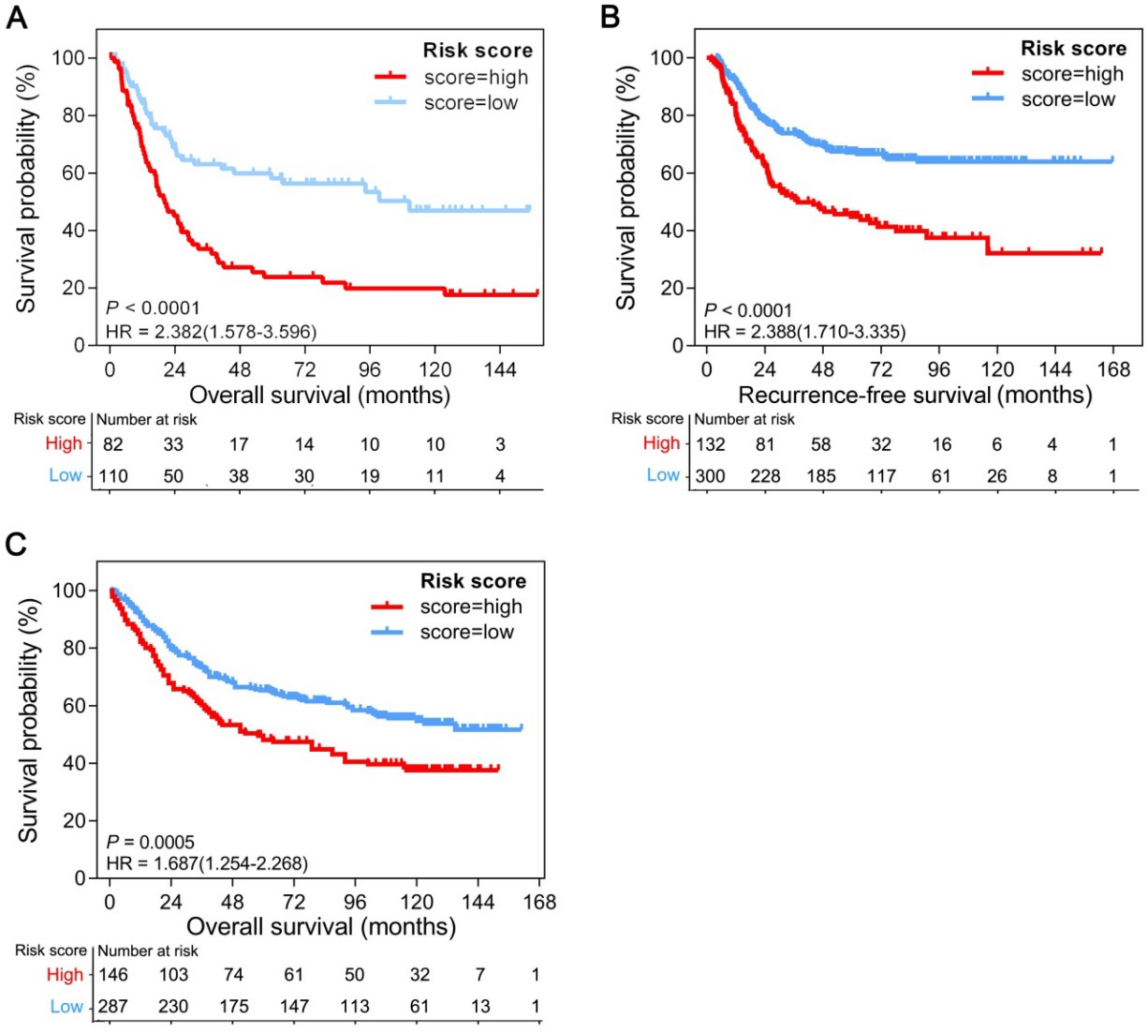
Kaplan-Meier curves of overall survival or recurrence-free survival according to GPSGC risk score in different gastric cancer validation cohorts. (**A**) GSE15459 (n = 192), (**B**) GSE26253 (n = 432), and (**C**) GSE84437 (n = 433). The provided *P* values are from log-rank tests. Patients were divided into high-risk and low-risk groups with GPSGC risk score = 0.15 utilized as the cutoff value.

To determine whether the GPSGC was robust, the performance of the GPSGC was assessed in three independent GEO GC cohorts, which totally consisted of 1057 GC patients. For the GSE15459 validation cohort, GPSGC successfully categorized 82 patients (42.7%) into the high-risk group and 110 patients (57.3%) into the low-risk group in terms of OS (HR = 2.382; 95% CI: 1.578-3.596; *P* < 0.0001; Figure 3A). Similar analyses showed that 132 high-risk patients (30.6%) had poorer recurrence-free survival (RFS) than 300 low-risk patients (69.4%) in the GSE26253 validation cohort (HR = 2.388; 95% CI: 1.710-3.335; *P* < 0.0001; Figure 3B), and 146 high-risk patients (33.7%) had poorer OS than 287 low-risk patients (66.3%) in the GSE84437 validation cohort (HR = 1.687; 95% CI: 1.254-2.268; *P* = 0.0005; Figure 3C). Consistent with the outcomes of the training cohorts, GC patients who were assigned to the high-risk group according to the GPSGC had significantly worse OS or RFS than those who were assigned to the low-risk group in multiple GEO validation cohorts.

### Association of TME subcomponents with GPSGC risk score and outcome in patients with gastric cancer

**Figure 4:**
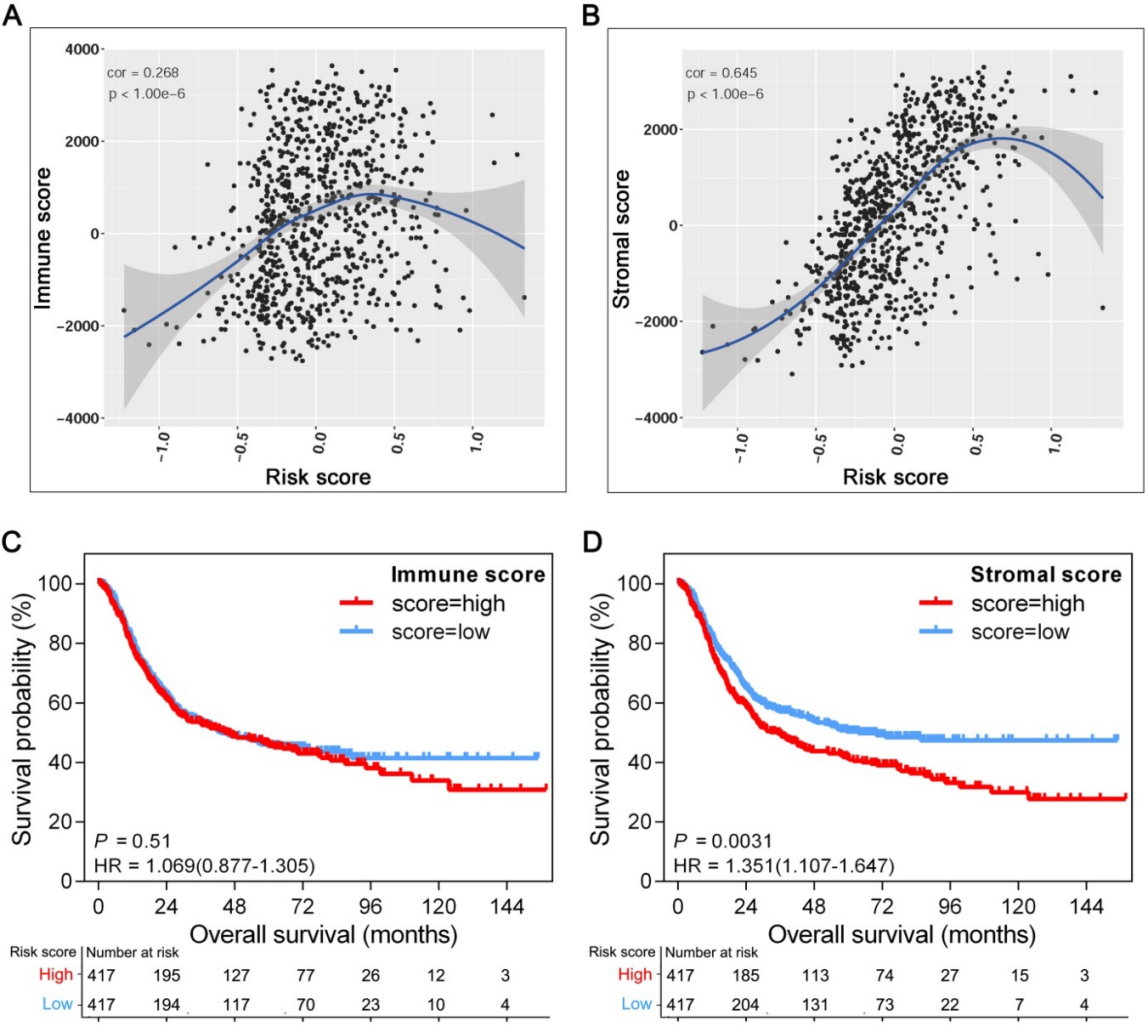
Association of TME subcomponents with GPSGC risk score and outcome in patients with gastric cancer. The 834 gastric cancer patients involved in the analysis were from the TCGA-STAD (n = 342), ACRG (n = 300) and GSE15459 (n = 192) cohorts. (**A**) Scatter plots depicting the low positive correlation between immune score and GPSGC risk score in human gastric cancer samples. The fitted curve of the relation between immune score and GPSGC risk score was obtained by locally weighted scatterplot smoothing (LOWESS). Pearson's correlation coefficient is shown in the graphs (*P* < 1.0×10^-6^). (**B**) Scatter plots depicting the strong positive correlation between stromal score and GPSGC risk score in human gastric cancer samples. The fitted curve of the relation between stromal score and GPSGC risk score was obtained by LOWESS. Pearson's correlation coefficient is shown in the graphs (*P* < 1.0×10^-6^). (**C**) Kaplan-Meier curves for overall survival of 834 gastric cancer patients according to immune score. Log-rank test, *P* = 0.51. (**D**) Kaplan-Meier curves for overall survival of 834 gastric cancer patients according to stromal score. Log-rank test, *P* = 0.0031.

The tissue distribution and cell location of VCAN, CLIP4 and MATN3 proteins suggest their relevance to the tumor microenvironment (TME) 27-29. Increasing evidence has elucidated the clinicopathological significance of TME characterization in predicting outcomes and therapeutic efficacy. To further explore the potential survival mechanisms related to the GPSGC, we first divided the TME into stromal and immune subcomponents based on the ESTIMATE algorithm and determined the stromal and immune scores by performing ssGSEA in the entire cohort of 834 patients (TCGA-STAD (n = 342), ACRG (n = 300), and GSE15459 (n = 192)). Pearson's correlation analysis revealed that the stromal score was strongly positively correlated with the GPSGC risk score in the entire cohort (r = 0.645; *P* < 1.0×10^-6^; Figure 4B); however, a low score was positively correlated with the immune score (r = 0.268; *P* < 1.0×10^-6^; Figure 4A). In addition, survival analysis was performed on the entire cohort of 834 patients with the median stromal score and median immune score utilized as the cutoff values. We found no significant difference in the OS of patients with high or low immune scores (HR = 1.069; 95% CI: 0.877-1.305; *P* = 0.51; Figure 4C), while GC patients with a high stromal score had worse overall survival than those with a low stromal score (HR = 1.351; 95% CI: 1.107-1.647; *P* = 0.0031; Figure 4D). Taken together, the above results suggest that the relationship of the GPSGC score with the poor survival of GC patients is probably related to the remodeling of stromal components in the tumor microenvironment.

### Association of TME cell types, panimmune gene sets and immunomodulatory genes with the GPSGC risk score and outcomes in patients with gastric cancer

**Figure 5:**
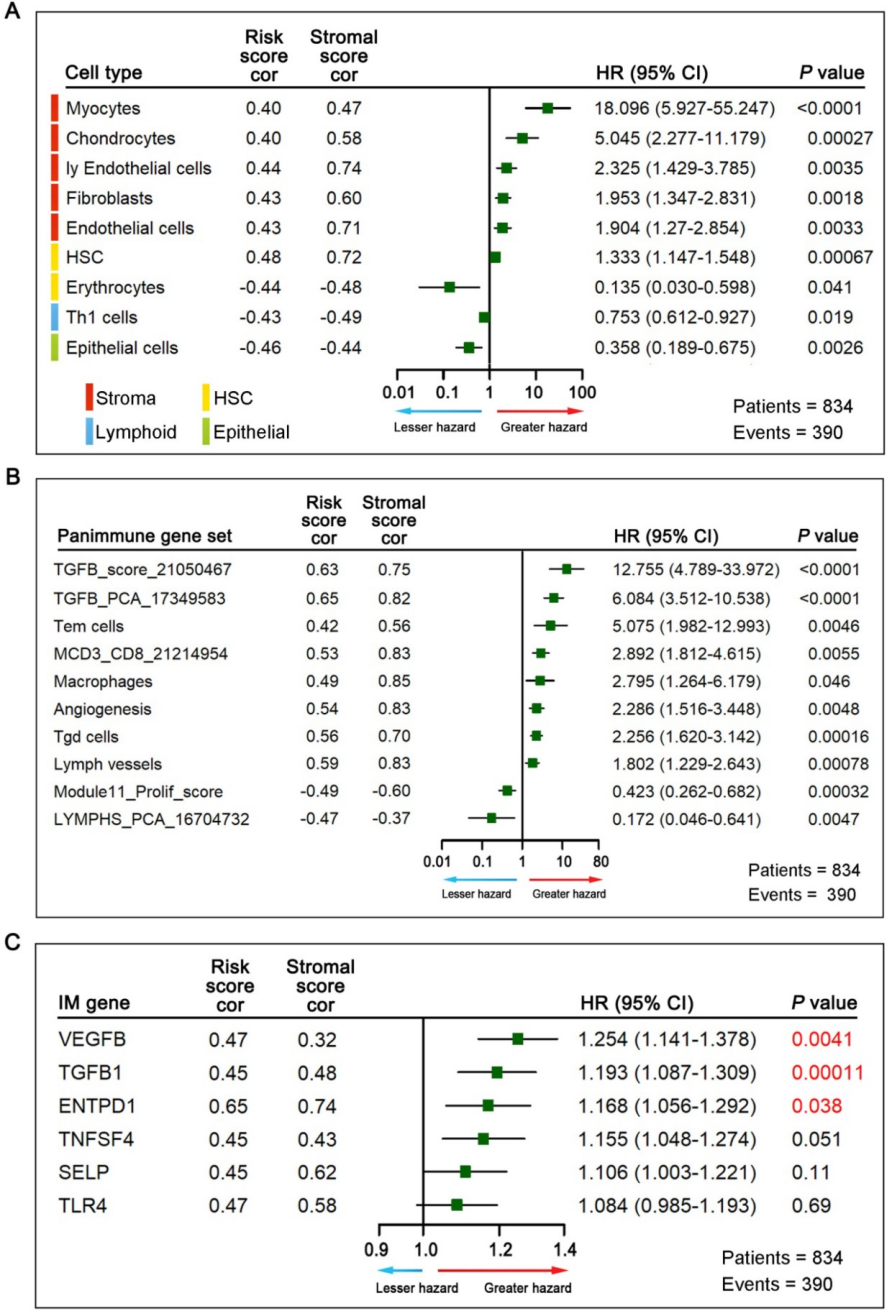
Association of TME cell types, panimmune gene sets, immunomodulatory (IM) genes with GPSGC risk score and outcome in patients with gastric cancer. The 834 gastric cancer patients involved in the analysis were from the TCGA-STAD (n = 342), ACRG (n = 300) and GSE15459 (n = 192) cohorts. (**A**) Among the 64 TME cell types, those significantly related to overall survival (log-rank test, *P* < 0.05) and GPSGC risk score (Pearson's correlation test, |r| ≥ 0.40, *P* < 0.05) are listed. The square data markers indicate estimated hazard ratios. The error bars represent 95% CIs. Pearson's correlation coefficients between 9 TME cell types and stromal scores are also shown (*P* < 0.05). (**B**) Among the 110 panimmune gene sets, those significantly related to overall survival (log-rank test, *P* < 0.05) and GPSGC risk score (Pearson's correlation test, |r| ≥ 0.40, *P* < 0.05) are listed. The square data markers indicate estimated hazard ratios. The error bars represent 95% CIs. Pearson's correlation coefficients between 10 panimmune gene sets and stromal scores are also shown (*P* < 0.05). (**C**) Among the 60 immunomodulatory genes, those significantly related to GPSGC risk score (Pearson's correlation test, |r| ≥ 0.40, *P* < 0.05) are listed. The overall survival analysis of 6 immunomodulatory genes is presented. The numbers marked in red denote estimates with a log-rank test *P*-value < 0.05. The square data markers indicate estimated hazard ratios. The error bars represent the 95% CIs. Pearson's correlation coefficients between the 6 immunomodulatory genes and stromal scores are also shown (*P* < 0.05).

To further elucidate relevant survival mechanisms related to the relationship between the GPSGC score and TME components and to explore GPSGC-related therapeutic targets, we carried out a series of TME characterization analyses at the cellular and molecular levels in the entire cohort of 834 patients (TCGA-STAD (n = 342), ACRG (n = 300), and GSE15459 (n = 192)). For TME cell type analysis, we inferred the proportions of 64 TME cell types based on the xCell algorithm and determined the enrichment score of each cell type by performing ssGSEA. Among the 64 TME cell types, those significantly related to OS (log-rank test, *P* < 0.05) and GPSGC risk score (Pearson's correlation test, |r| ≥ 0.40, *P* < 0.05) are listed in Figure 5A, yielding a total of 9 cell types. Remarkably, all the five types of stromal cells with the largest proportions were positively correlated with prognosis and GPSGC risk score. With the second highest proportions were two types of cells belonging to hematopoietic stem cells (HSCs), and one of them were positively associated with poor outcome and GPSGC risk score. In addition, there were one type of lymphoid cells and one type of epithelial cells, both of which were negatively associated with poor prognosis and GPSGC risk score.

Next, we explored potential GPSGC-associated therapeutic targets through TME characterization analysis at the molecular level. For panimmune gene set analysis, gene set variation analysis (GSVA) was used to estimate the enrichment scores of 110 immunoregulation-related pathways in the entire cohort of 834 GC patients. Gene sets that were significantly associated with OS (log-rank test, *P* < 0.05) and GPSGC risk score (Pearson's correlation test, |r| ≥ 0.40, *P* < 0.05) we screened out, resulting in 10 panimmune gene sets, 8 of which were positively associated with poor outcome and GPSGC risk score (Figure 5B). Interestingly, TGFβ-related gene sets and angiogenesis-related gene sets closely associated with the remodeling of stromal components in the TME were highlighted in this screening. To identify GPSGC-associated specific molecular targets, 60 detectable immunomodulatory genes were analyzed in the entire cohort of 834 GC patients and were significantly correlated with the GPSGC risk score (Pearson's correlation test, |r| ≥ 0.40, *P* < 0.05); the genes are listed in Figure 5C. OS analysis further showed that only three immunomodulatory genes, VEGFB, TGFβ1 and ENTPD1, were significantly associated with poor outcomes. Taken together, the above results highlight the potential survival mechanisms and therapeutic targets related to the GPSGC.

### Experimental verification of the GPSGC and therapeutic targets in GC tissue microarrays

**Figure 6:**
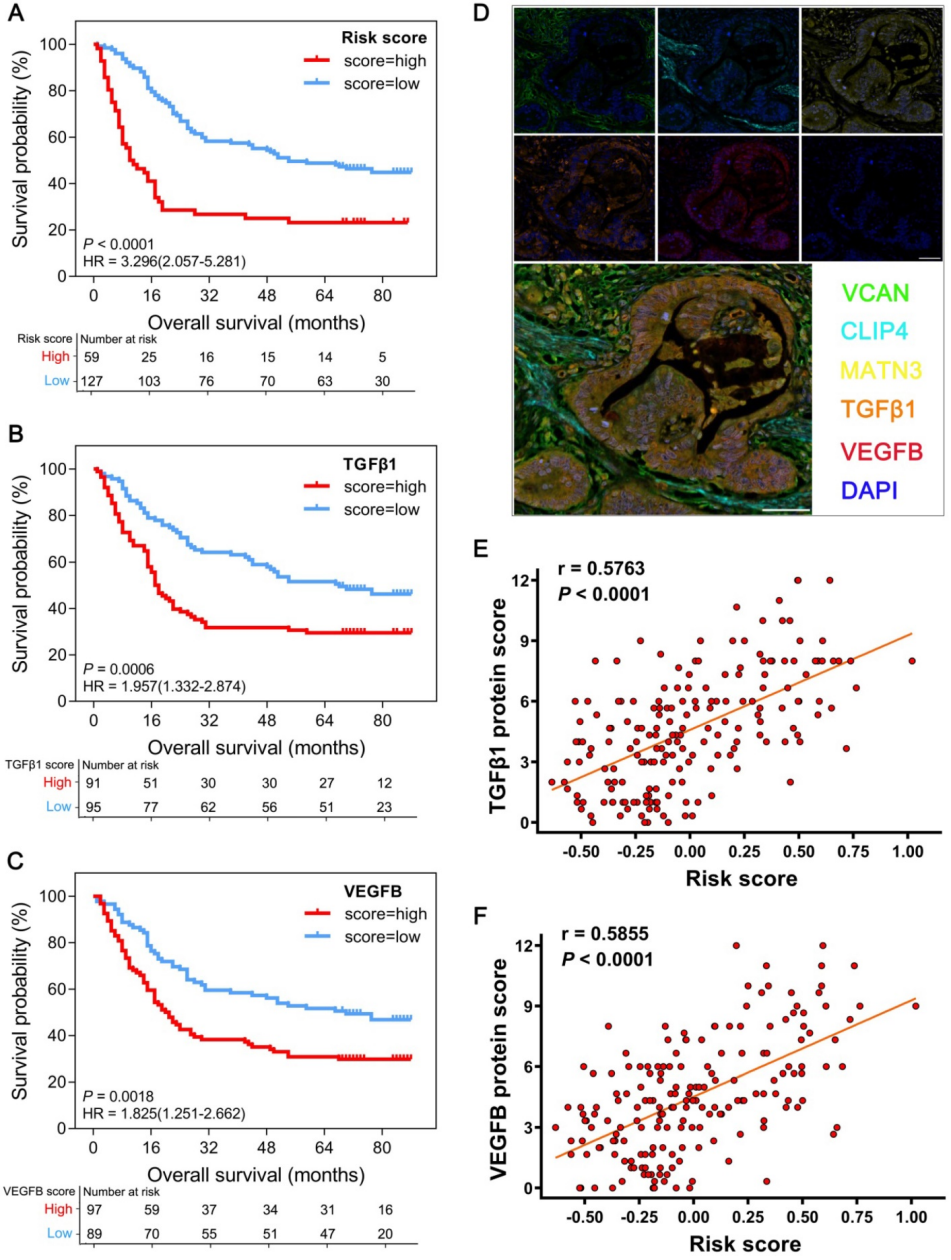
Multiplex fluorescent immunohistochemistry (mfIHC) analysis of the relationship between GPSGC risk score, therapeutic target expression and overall survival with GC tissue microarray data. (**A**) Kaplan-Meier curves for high (n = 59) and low (n = 127) GPSGC risk score patient groups in GC tissue microarray data. Log-rank test, *P* < 0.0001. (**B**) Kaplan-Meier curves for high (n = 91) and low (n = 95) TGFβ1 expression patient groups in GC tissue microarray data. Log-rank test, *P* = 0.0006. (**C**) Kaplan-Meier curves for high (n = 97) and low (n = 89) VEGFB expression patient groups in GC tissue microarray data. Log-rank test, *P* = 0.0018. (**D**) mfIHC showed the protein expression and localization of VCAN (green), CLIP4 (cyan), and MATN3 (yellow) and the therapeutic targets TGFβ1 (orange) and VEGFB (red) in GC tissue. DAPI: blue; scale bar: 50 µm. (**E**) Scatter plots depicting the positive correlation between GPSGC risk score and TGFβ1 expression in GC tissue microarray data. Pearson's correlation coefficient is shown in the graphs (n = 186, *P* < 0.0001). (**F**) Scatter plots depicting the positive correlation between GPSGC risk score and VEGFB expression in GC tissue microarray data. Pearson's correlation coefficient is shown in the graphs (n = 186, *P* < 0.0001).

As all the proteins associated with the GPSGC and therapeutic targets perform important biological functions, we further used GC tissue microarrays combined with multiplex fluorescent immunohistochemistry (mfIHC) for experimental verification at the protein level. In tissue microarrays from 186 GC patients, the protein expression-modified GPSGC effectively categorized 59 patients (31.7%) into the high-risk group and 127 patients (68.3%) into the low-risk groups in terms of OS (HR = 3.296; 95% CI: 2.057-5.281; *P* < 0.0001; Figure 6A). OS analysis also proved that GC tissue protein expression of the therapeutic targets TGFβ1 and VEGFB was significantly associated with poor outcome of the 186 GC patients (Figure 6B-C). As indicated by the representative GC sample in the tissue microarrays in Figure 6D, the overall expression levels and localization of VCAN, CLIP4 and MATN3 were dramatically correlated with the expression of the therapeutic targets TGFβ1 and VEGFB. Pearson's correlation analysis revealed that the protein expression-modified GPSGC risk score was strongly positively correlated with TGFβ1 protein expression (r = 0.5763; *P* < 0.0001; Figure 6E) and VEGFB protein expression (r = 0.5855; *P* < 0.0001; Figure 6F) in the tissues of 186 GC patients. Together, these results experimental verified the stability and reliability of the GPSGC model, which further suggested that TGFβ1 and VEGFB may be developed as potential therapeutic targets for GC patients with poor prognosis according to the GPSGC.

### Construction and evaluation of a nomogram based on the GPSGC

**Figure 7:**
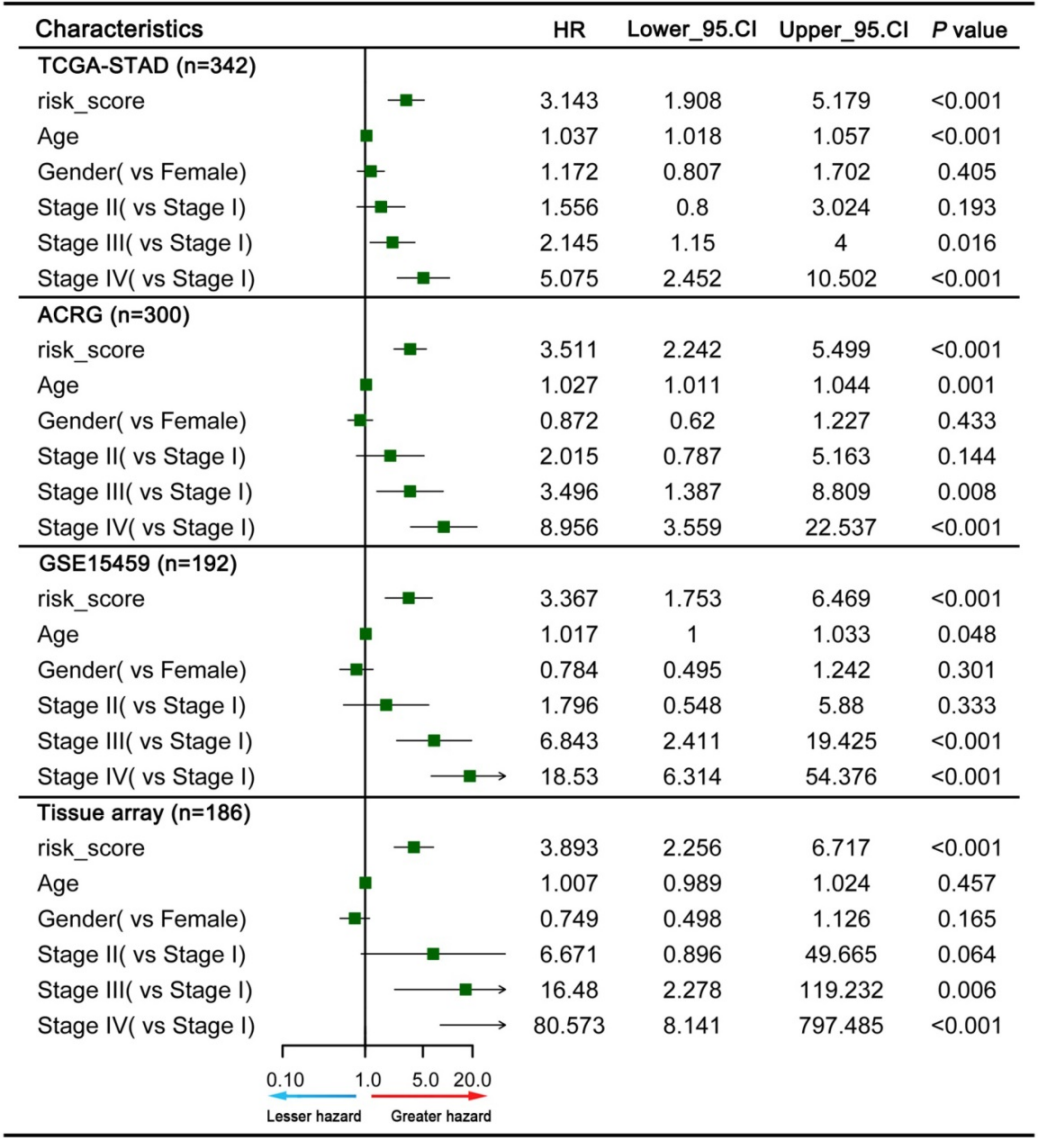
Multivariate Cox analysis evaluating independently predictive ability of the GPSGC and other clinical risk factors for OS. The square data markers indicate estimated hazard ratios. The error bars represent 95% CIs.

**Figure 8:**
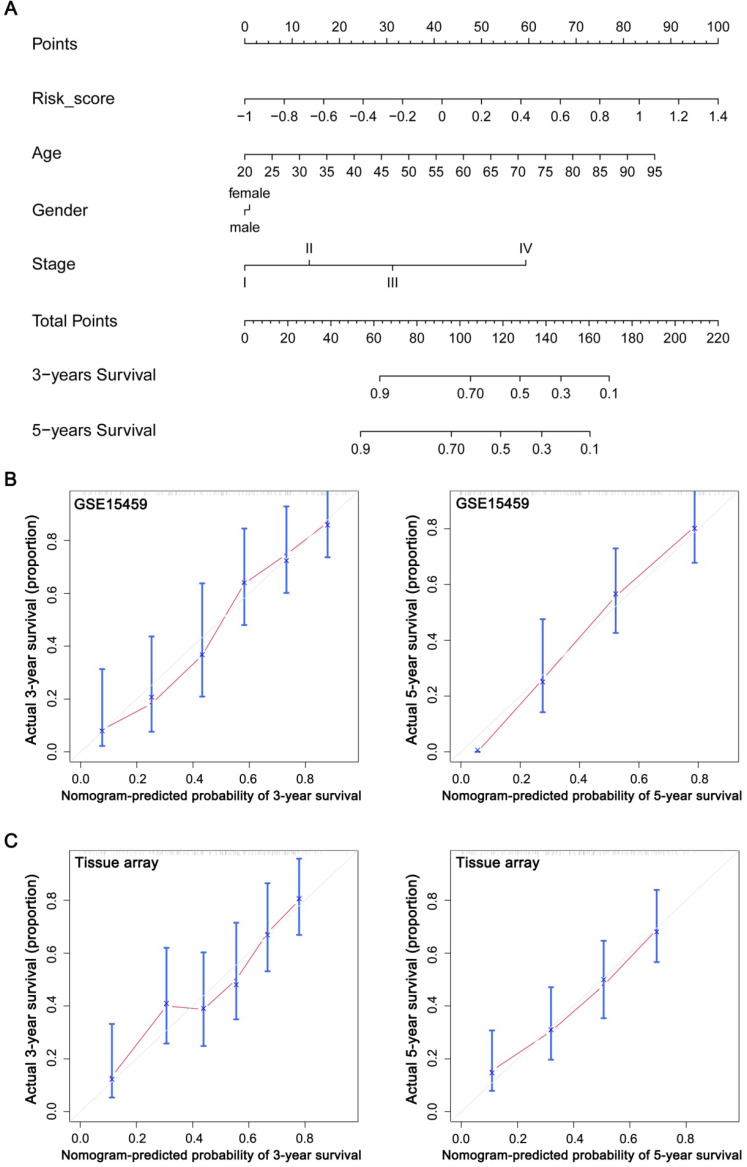
Construction and evaluation of a nomogram based on the GPSGC to predict the 3-year and 5-year overall survival for GC patients. (**A**) Nomogram was constructed with the TCGA-STAD and ACRG training cohorts (n = 642) for predicting the probability of 3-year and 5-year OS for GC patients. (**B**) Calibration plot of the nomogram for predicting the probability of OS at 3, and 5 years in GSE15459 validation cohort (n = 192). (**C**) Calibration plot of the nomogram for predicting the probability of OS at 3, and 5 years in the experimental tissue array validation cohort (n = 186). The grey line represents the ideal nomogram, and the red line represents the observed nomogram.

To explore whether the prognostic value of the GPSGC was independent of other clinical factors, multivariate Cox regression analyses were conducted, which exhibited that GPSGC could serve as an independent predictor of patients' survival outcome after adjusted by clinical characteristics including age, gender, and AJCC stage in multiple GC cohorts, thus confirming its robustness for independently predicting GC prognosis (Figure 7). Based on multivariate Cox analysis, a nomogram that integrated the GPSGC and other clinical variables was generated to predict the probability of 3-year and 5-year overall survival for GC patients with the TCGA-STAD and ACRG training cohorts (Figure 8A). The calibration plots for the probability of OS at 3, and 5 years were predicted well in the GSE15459 validation cohort (C-index 0.754, 95% CI 0.709-0.798; Figure 8B), and the experimental tissue array validation cohort (C-index 0.706, 95% CI 0.653-0.759; Figure 8C).

## Discussion

Patients suffering from GC often display heterogeneous clinical outcome, with survival durations ranging from less than 5 months to over 10 years 4, 11. The 5-year overall survival rate of patients with early-stage localized GC is more than 60%, whereas that of patients with distant metastasis is less than 5% 30. When diagnosed at early stages, GC can be effectively treated with endoscopic or surgical resection with or without adjuvant therapy. However, survival outcomes can vary widely among patients receiving the same treatment for disease of the same stage 31. In this respect, treatment options are lacking, with all patients being treated with similar drugs. Thus, a prognostic signature beyond the current staging system is desired to accurately identify those patients likely to develop refractory disease and have worse survival and to better guide adjuvant treatment. At present, many independent studies have found that some gene transcription levels are closely related to the outcome of gastric cancer 14, 29, 32, 33, but there have been a lack of comprehensive bioinformatic, clinicopathological factors and machine learning analyses to improve the accuracy of prognosis. In this study, using multiple well-established public gastric cancer cohorts, we developed a robust prognostic signature on the basis of gene set enrichment analysis and proved its efficacy in three GEO datasets derived from different microarray platforms. Furthermore, experimental verification was carried out to prove the GPSGC model stability and reliability at the protein level using tissue microarray data from 186 GC patients. To provide clinicians with a quantitative approach to predict the prognosis of GC patients, a nomogram that integrated the GPSGC and other clinical variables was constructed, which is more accurate for predicting short-term and long-term survival in GC patients than individual prognostic factors.

The three genes used to assess GPSGC risk scores in our study (VCAN, CLIP4 and MATN3) have been previously reported to be associated with gastric cancer. VCAN (versican), a ubiquitous component of the extracellular matrix (ECM), accumulates in both tumor stroma and cancer cells and is highly regulated by various cytokines 27. Many investigators have proven that high expression of VCAN is an independent predictor of poor prognosis in gastric cancer and correlates with advanced stage and T classification 34, 35. Furthermore, abnormally expressed VCAN functionally participates in the progression of gastric cancer 27. CLIP4, also known as UBASH3A or TULA, is a member of the T cell ubiquitin ligand family 28. Some studies have shown that CLIP4 expression stimulates tumor metastasis and recurrence in certain tumor types, and its promoter methylation is associated with an increase in GC severity 36-38. MATN3 (matrilin-3) is widely considered to be involved in the formation of filamentous networks in the extracellular matrix of various tissues 39. Previous studies have found that MATN3 mRNA and protein are highly expressed in GC patients, and MATN3 overexpression could be used as an independent predictor of poor prognosis in GC patients 29, 40. Although these three genes were previously found to be related to the prognosis of gastric cancer, our study is the first to our knowledge to report the feasibility and accuracy of a risk assessment model based on VCAN, CLIP4 and MATN3 expression for determining GC prognosis.

The tissue distribution and cell localization of the VCAN, CLIP4 and MATN3 proteins suggest their relevance to the tumor microenvironment (TME). Moreover, the GPSGC risk score was strongly positively correlated with the stromal score and weakly positively correlated with the immune score in the TME subcomponent analysis of our study, while a high stromal score was associated with poor survival of GC patients, and the immune score was not related to survival. Therefore, we infer that the effect of VCAN, CLIP4 and MATN3 expression on the poor survival of GC patients is probably related to the remodeling of stromal components in the tumor microenvironment. Our TME cell type analysis also fully supports this deduction. Studies have shown that the abundance of stromal components as an independent prognostic factor was critical to prognostication in GC 31, 41, which also provide indirect evidence for the close relationship between GPSGC and survival of GC patients. Taken together, these results provide new insights for cell omics research on the mechanism by which VCAN, CLIP4, and MATN3 regulate the survival of GC patients.

To explore potential therapeutic targets for gastric cancer patients with poor prognosis based on the GPSGC, we further performed panimmune gene set analysis and immunomodulatory gene analysis using gene expression data of GC. The results of the panimmune gene set analysis showed that the TGFβ-related gene set and the angiogenesis-related gene set were significantly correlated with the GPSGC risk score and poor survival. In addition, the immunomodulatory gene analysis accurately indicated that TGFβ1 and VEGFB may be developed as potential therapeutic targets of GC patients with poor prognosis according to the GPSGC. Our experimental verification in GC tissue microarrays also confirmed this conclusion at the protein level. At present, galunisertib and M7824 are targeted drugs that have been used in the clinical treatment of gastric cancer and key function by blocking the TGFβ signaling pathway 42-45. There is increased expression of VEGFB in gastric cancer, and clinical drugs such as apatinib and Zaltrap can effectively block its function 46-50. Therefore, these drugs targeting TGFβ1 or VEGFB may be developed to be combined with our prognosis signature to achieve accurate treatment for GC patients.

Our study has several strengths. First, the GC cohorts had large sample sizes and were systematically analyzed in multiple ways in this study. All detectable genes were included in the analysis, and machine learning was used to explore the optimal prognosis model. Second, we further performed a comprehensive TME characterization analysis to explore the GPSGC-related survival mechanisms and potential therapeutic targets of GC. Moreover, we carried out experimental verification to prove the GPSGC model stability and reliability at the protein level using tissue microarray data, and a significant correlation between GPSGC risk score and the protein expression of therapeutic targets was identified.

Despite the significant results obtained in the present study, there were several shortcomings. First, although large sample-sized GC cohorts were included in our study to establish a well-validated prognostic model, sampling bias due to the use of different platforms may result in some subjectivity of the gene expression values. Second, our study provided new insights into the GC stromal microenvironment and related therapy targets. However, it has limitation because it was retrospective. Thus our findings should be further confirmed by prospective studies. Third, the biological mechanisms by which the three genes (VCAN, CLIP4 and MATN3) integrated into the GPSGC model in our study contribute to GC progression and poor survival remain elusive, and further in-depth investigations into their functions might provide novel targets and treatment strategies.

In conclusion, this study identified a TME-relevant gene set-based prognostic signature that can effectively predict GC patient survival outcomes. The GPSGC model can be clinically used to improve GC patient OS and to develop individualized therapy based on GPSGC-related targeted drugs.

## Supplementary Material

Supplementary figures.

Supplementary table 1.

Supplementary table 2.

Supplementary table 3.

Supplementary table 4.

Supplementary table 5.

Supplementary table 6.

Supplementary table 7.

## Abbreviations

AI: artificial intelligence
DEGs: differentially expressed genes
EBV: Epstein-Barr virus
ECM: extracellular matrix
FPKM: fragments per kilobase million
GC: gastric cancer
GEO: Gene Expression Omnibus
GPSGC: gene-expression-based prognostic signature for gastric cancer
GSVA: gene set variation analysis
HSC: hematopoietic stem cell
KM: Kaplan-Meier
LOWESS: locally weighted scatterplot smoothing
mfIHC: multiplex fluorescent immunohistochemistry
OS: overall survival
RFS: recurrence-free survival
TCGA: The Cancer Genome Atlas
TME: tumor microenvironment
TPM: transcripts per kilobase million
