# Supplementary material for: Identification of a Tumor Microenvironment-relevant Gene set-based Prognostic Signature and Related Therapy Targets in Gastric Cancer
Source: Theranostics. 2020 Jul 9;10(19):8633–47. doi: 10.7150/thno.47938 (PMC7392024; doi:10.7150/thno.47938)
Supplement: Supplementary file 1 — Supplementary figures. [file thnov10p8633s1.pdf]

Supplementary figure

Figure S1

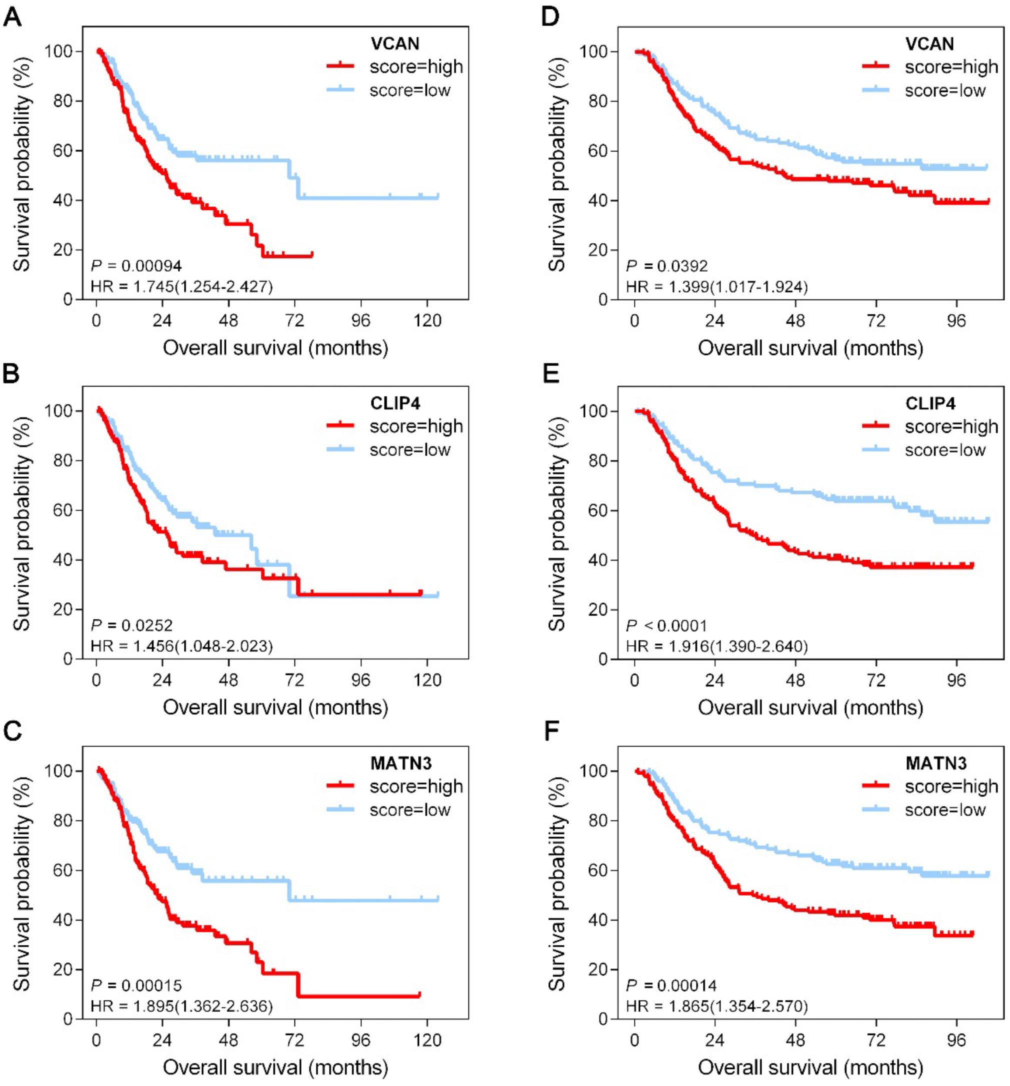

Figure S1. Kaplan–Meier curves for overall survival in the TCGA-STAD and ACRG cohorts according to the expression of individual prognostic genes. (A-C) Kaplan–Meier curves for overall survival of 342 gastric cancer patients from the TCGA-STAD cohort according to the VCAN mRNA level (A), CLIP4 mRNA level (B), and MATN3 mRNA level (C). The provided *P* values are from log-rank tests. (D-F) Kaplan–Meier curves for overall survival of 300 gastric cancer patients from the ACRG cohort according to the mRNA level of VCAN (D), CLIP4 (E), or MATN3 (F). The provided *P* values are from log-rank tests.
